# Prompt engineering for single-best-answer multiple-choice questions in licensing examinations: a narrative review with a case study involving the Korean Medical Licensing Examination

**DOI:** 10.3352/jeehp.2025.22.34

**Published:** 2025-10-27

**Authors:** Bokyoung Kim, Junseok Kang, Min-Young Kim, Jihyun Ahn

**Affiliations:** 1 College of Nursing, Research Institute of Nursing Innovation, Kyungpook National University, Daegu, Korea; 2 Harvard John A. Paulson School of Engineering and Applied Sciences, Harvard University, Cambridge, MA, USA; 3 Department of Dental Hygiene, Howon University, Gunsan, Korea; 4 Department of Internal Medicine, Korea Medical Institute, Seoul, Korea; The Catholic University of Korea, Korea

**Keywords:** Artificial intelligence, Large language models, Medical licensure, Multiple-choice questions, Republic of Korea

## Abstract

The emergence of large language models (LLMs) has generated growing interest in their potential applications for medical assessment and item development. This practice-oriented narrative review examines the potential of LLMs, particularly ChatGPT, for generating and validating single-best-answer multiple-choice questions in health professions licensing examinations, using a Korean Medical Licensing Examination (KMLE)-focused case perspective. We frame LLMs as human-in-the-loop tools rather than replacements for high-stakes testing. Recent applications of LLMs in assessment were reviewed, including prompting strategies such as few-shot, multi-stage, and chain-of-thought methods, as well as retrieval-augmented generation (RAG) to align outputs with exam blueprints. Approaches to enforcing formatting rules, checklist-based self-validation, and iterative refinement were analyzed for their role in supporting item development. Findings indicate that LLMs can perform near passing thresholds on high-stakes exams and assist with grading and feedback tasks. Prompt engineering enhances structural fidelity and clinical plausibility, while human oversight remains critical for accuracy, cultural appropriateness, and psychometric defensibility. The emerging multimodal generation of images, audio, and video suggests the feasibility of new item formats, provided robust validation safeguards are implemented. The most effective approach is a human-in-the-loop workflow that leverages artificial intelligence efficiency while embedding expert judgment, psychometric evaluation, and ethical governance. This practice-oriented roadmap—integrating strategic prompt selection, RAG-based blueprint alignment, rigorous validation gates, and KMLE-specific formatting—offers an implementable and methodologically defensible approach for licensing examinations.

## Graphical abstract

[Fig f3-jeehp-22-34]

## Introduction

The rapid emergence of large language models (LLMs), exemplified by ChatGPT, has attracted substantial attention in education, research, and assessment within the health professions. Since its release in late 2022, ChatGPT has demonstrated a wide range of applications, from essay generation and programming support to clinical education and biomedical research [1]. Its conversational and generative capabilities distinguish it from traditional information retrieval tools. In this article, we explicitly position LLMs not as replacements for high-stakes testing but as human-in-the-loop tools that support safe, auditable item development workflows, thereby clarifying the scope and intent of this work from the outset. Alongside these developments, several recent reviews have examined LLM-based multiple-choice question (MCQ) generation and broader assessment applications, documenting both promising feasibility and critical limitations while underscoring the need for rigorous validation protocols [2-4].

Recent evaluations suggest that ChatGPT’s performance on medical licensing assessments is approaching, and in some domains surpassing, the passing threshold. Such performance, however, is highly dependent upon task characteristics, subject domain, language and translation quality, and item format, and therefore should not be generalized across examinations without careful consideration. In Korea, ChatGPT scored approximately 60% on a standardized parasitology test, a level comparable to that of medical students [5]. In Japan, GPT-4 successfully completed the national pharmacist licensing examination, including items requiring diagram interpretation [6]. Comparable outcomes have been reported in Peru [7] and Thailand [8], although performance varied by subject area and translation fidelity. Beyond answering items, GPT-4 has also shown potential in assessment tasks, such as grading pharmacy students’ written responses, achieving agreement rates comparable to expert raters in Malaysia [9].

Despite these advances, scholars caution that the direct use of LLMs in high-stakes testing raises serious concerns regarding reliability and validity [10,11]. A promising alternative is the use of LLMs as tools for automatic item generation (AIG). Studies in pharmacotherapy, surgery, and medical education demonstrate that LLM-based AIG can accelerate test development and reduce faculty workload [12]. However, ensuring quality—particularly the plausibility of distractors, realism of clinical vignettes, and strict adherence to examination formatting rules—remains essential for validity [13]. Accordingly, we adopt a validation-first approach in which no generated item proceeds to item banking without passing rigorous expert review and automated quality checks; this principle directly informs the workflow and guidance presented in this review.

Among MCQ formats, the single-best-answer (SBA, or A-type) item predominates in licensing examinations, including the United States Medical Licensing Examination (USMLE), the Korean Medical Licensing Examination (KMLE), and similar assessments worldwide [14]. SBA items follow established conventions: a clinical vignette leading to a focused question, 5 options with 1 correct answer, and distractors that are plausible yet incorrect. In Korea, the KMLE imposes additional requirements concerning vignette sequencing, option presentation, and laboratory reference notation [15]. Because practical adoption depends critically on compliance with these conventions, our review emphasizes format fidelity and regulatory alignment through KMLE-specific illustrations rather than generic prompting approaches.

To align ChatGPT outputs with such standards, prompt engineering has become a crucial strategy. Zero-shot prompts may produce creative but inconsistent results, whereas few-shot prompting with authentic exemplars improves format adherence and distractor plausibility [12]. We also examine chain-of-thought (CoT) prompting, which decomposes question generation into transparent intermediate steps and has been shown to enhance few-shot performance when prompts explicitly scaffold sub-tasks [16], while ensuring that rationales remain hidden from learner-facing materials. Advanced methods such as multi-stage or CoT prompting improve logical consistency and clinical accuracy [17,18], while retrieval-augmented generation (RAG) enables alignment with curricular blueprints and mitigates content drift [19].

Although several recent reviews have analyzed generative artificial intelligence (GenAI) applications in assessment and MCQ generation, spanning both theoretical surveys and practitioner-focused syntheses [2-4], the present narrative review makes a distinctive, practice-oriented contribution. It synthesizes prompt selection guidance for end users, maps risk points to validation checkpoints, and provides KMLE-aligned exemplars and checklists that educators and psychometricians can apply immediately. Our approach differs from existing reviews by emphasizing practical implementation in licensing examination contexts—specifically through RAG-driven blueprint alignment methodologies, validation-first governance frameworks, and jurisdiction-specific formatting specifications that enable institutions to establish defensible artificial intelligence (AI)-assisted workflows for immediate adoption [2-4].

This narrative review integrates recent developments in prompt engineering for SBA MCQ generation in health professions licensing examinations, with particular focus on the KMLE. By combining international evidence with Korean regulatory requirements, we aim to provide a comprehensive roadmap that is both practically implementable and methodologically defensible—one that leverages GenAI capabilities to enhance item development while maintaining validity, fairness, and cultural appropriateness.

## Methods

This narrative review draws upon recent literature examining LLM applications in medical assessment, with an emphasis on SBA item generation for licensing examinations. Our synthesis incorporates studies and reports published between January 2022 and September 2025, identified through searches of PubMed, Google Scholar, IEEE Xplore, and leading medical education journals. We contextualized these findings within the KMLE framework by referencing publicly available item development guidelines and KMLE-specific requirements.

## LLM performance in licensing examinations: setting the context

The first wave of studies on LLMs such as ChatGPT has investigated their ability to solve existing items from national licensing examinations. These findings provide a useful benchmark for assessing their potential role in high-stakes medical testing and item generation. It is important to note from the outset that such benchmarks are highly context-dependent, as performance varies substantially across domains, languages, translation quality, item formats, and scoring methodologies. Therefore, these results should be interpreted cautiously when extrapolating to item development applications.

### Evidence from Korea and the United States

In Korea, GPT-based models have recently been benchmarked against the KMLE, achieving performance close to or above the passing threshold in several domains [20]. However, results vary considerably across subjects and test years, appearing sensitive to both prompt design choices and translation quality. Comparable passing-level outcomes have also been reported in other Korean health professions licensing tests, including those in nursing and dentistry, while ChatGPT achieved passing scores in the Korean oriental medicine (Korean medicine) licensing examination [21]. In the United States, GPT-4 demonstrated passing or near-passing performance across all 3 steps of the USMLE. Nevertheless, concerns about item exposure, contamination risks, and evolving test blueprints preclude treating these results as indicators of generalized capability [22].

### International evidence

Beyond Korea and the United States, ChatGPT and related models have produced promising results elsewhere. In Peru, ChatGPT performed competitively with human examinees on the national medical licensing examination [7]. In Thailand, LLMs reached or closely approximated the cut score on the Thai National Medical Licensing Examination [8]. Complementary findings also emerge from other health professions: in Japan, GPT-4 successfully passed the pharmacist licensing examination, including items requiring diagram interpretation [6]. Although these results extend beyond physician licensing, they confirm the adaptability of LLMs to discipline-specific assessments in both basic and applied sciences. Collectively, these studies highlight contextual capability rather than universal readiness across all licensing environments.

### Beyond answering: grading and evaluation

The utility of LLMs extends beyond answering test questions. In Malaysia, GPT-4 was used to grade pharmacy students’ short-answer responses and demonstrated reliability comparable to that of expert assessors [9]. These findings suggest potential applications in triage, formative feedback, and rubric-aligned scoring—provided that robust human oversight and comprehensive audit mechanisms are maintained, highlighting the potential for LLMs to support not only item generation but also grading and feedback functions in health professions education.

### Implications for item generation

Taken together, these findings indicate that LLMs can engage effectively with examination content across diverse professional domains and national settings. However, item generation poses qualitatively distinct challenges. Unlike answering, item development requires strict adherence to psychometric standards, the construction of plausible distractors, and compliance with exam-specific formatting conventions [11,13]. Accordingly, we adopt a validation-first framework in which all LLM outputs must undergo rigorous content-expert review, automated quality checks (including detection of option–key conflicts, cueing, and duplication), and pilot testing before any item enters the item bank. Performance benchmarks on licensing exams thus establish a foundation but do not obviate the need for advanced prompt-engineering techniques (such as few-shot and CoT methodologies during authoring, with rationales appropriately suppressed in learner-facing materials) and governance frameworks aligned with local regulatory requirements (such as KMLE formatting specifications).

## Prompting strategies for SBA MCQ generation

The quality of AI-generated MCQs depends heavily on the prompting strategies employed. While LLMs such as ChatGPT possess extensive medical knowledge, their outputs vary substantially depending on how tasks are framed. For SBA items, adherence to strict structural and psychometric rules requires deliberate prompt engineering. This section serves as a practical selection guide for practitioners, systematically mapping prompting choices to end-user objectives while explicitly linking them to essential validation gates—including expert review, automated quality checks, and pilot statistics—that must be completed before any item enters the item bank (Table 1).

### Zero-shot prompting: strengths and weaknesses

Zero-shot prompting, in which the model is asked to generate questions without prior examples, offers the advantages of simplicity and speed. It can produce diverse question types spanning recall, problem-solving, and clinical reasoning domains [23]. However, studies consistently show that zero-shot outputs often deviate from required formats, yielding implausible distractors, inconsistent option ordering, or ambiguity [24]. In licensing contexts—where reliability, fairness, and standardization are paramount—these weaknesses limit the utility of zero-shot methods. In practice, we recommend employing zero-shot prompting exclusively during the initial ideation phase, followed immediately by systematic filtering using structural checklists (ensuring single best answers, unambiguous lead-ins, and option homogeneity) and automated cueing detection (screening for option-length bias and key-position bias). Only items that pass these preliminary filters should advance to expert review.

### Few-shot prompting with authentic exemplars

Few-shot prompting supplies the model with exemplar questions before generation. This approach has been shown to markedly improve structural conformity and consistency in generated items [25]. In medical education, providing well-crafted case vignettes as exemplars enables LLMs to reproduce the tone, length, and reasoning patterns found in validated examinations [12]. For licensing applications, we have observed that 3 to 5 high-quality exemplars covering the target blueprint domain yield optimal results. Each exemplar should be clearly formatted with explicit tags for stems, options A–E, correct answers, and hidden rationales to ensure consistent output structure. When exemplars reflect local conventions—such as the KMLE’s standardized order of demographics, chief complaint, history and examination findings, investigations, and vital signs—the model demonstrates significantly improved format fidelity and generates culturally and linguistically appropriate content [14,15,26].

### Multi-stage prompting for decomposed item generation

Multi-stage prompting generates items step by step, beginning with the topic or competency area and proceeding through the vignette, stem, correct answer, and explanatory rationale. This decomposition reduces logical errors and minimizes the risk of answer leakage into the vignette [23]. Recent work on self-refining item generation further suggests that multi-stage workflows allow iterative correction, enabling the model to review and amend its own output until it meets structural checklist criteria [27]. In practice, CoT prompting operationalizes this decomposition by selecting supportive exemplars and defining sub-steps explicitly, thereby improving coherence and fidelity under few-shot constraints [16]. We recommend a 6-stage process: (1) establishing the topic and blueprint alignment; (2) drafting the clinical vignette without options; (3) formulating a focused lead-in question; (4) developing 5 balanced options with 1 defensible correct answer; (5) creating hidden rationales with supporting citations and decision logs; and (6) performing systematic self-critique to identify failure modes such as distractor implausibility, unintended cueing, or multiple defensible answers. Only items that successfully pass all 6 stages should proceed to human expert review and small-scale pilot testing.

### Structured prompt formats and custom GPTs

Recent research shows that structured formats such as markdown and JavaScript Object Notation (JSON) substantially improve the clarity and accuracy of LLM outputs compared with plain-text prompts. A study on the Gemini LLM found that JSON-based prompting improved accuracy by 21.62 percentage points, highlighting the efficiency of structured schemas such as YAML and JSON [28]. Another investigation reported that markdown prompts using headings, lists, and bold markers enhanced models’ ability to capture information hierarchies and produce more systematic responses [29]. Following established vendor best practices, we employ JSON schemas to enforce consistent field structures (including blueprint alignment, vignettes, lead-ins, multiple options, correct answers, and hidden rationales) while using Markdown formatting to communicate human-readable evaluation rubrics and quality checklists. Importantly, rationales are always suppressed in learner-facing materials to prevent unintended cueing or content leakage.

Beyond formatting, workflows can be streamlined by developing custom GPTs that encapsulate role instructions, curated data sources, and reusable prompts within a single deployable model. Evidence suggests that custom GPTs can serve as administrative assistants, course tutors, or virtual patients, making them particularly valuable for health professions education [30]. This approach reduces repetitive prompt engineering and facilitates secure, learner-friendly deployment. For licensing preparation and faculty authoring contexts, custom GPTs can be configured to embed blueprint alignment protocols, format specifications (such as KMLE requirements), and distractor-development policies from the outset. These systems can also log retrieval sources when RAG is employed and provide integrated 1-click validation tools, allowing users to maintain consistency without re-entering detailed instructions (Table 2, Supplement 1).

## RAG for blueprint and curriculum alignment

One of the most persistent challenges in AI-based item generation is ensuring that questions align with the test blueprint and official curriculum. Without explicit constraints, LLMs may produce items that are clinically accurate but irrelevant to the intended examination scope. RAG addresses this limitation by grounding prompts in authoritative external documents such as exam blueprints, learning objectives, and guideline repositories. In this review, we conceptualize RAG not merely as a modeling technique but as a foundational governance mechanism, wherein every generated item must include comprehensive retrieval logs documenting sources, sections, and timestamps. Each item must also successfully pass content-expert verification before advancing to the item bank.

### Blueprint-driven coverage

Licensing examinations such as the KMLE and USMLE are constructed according to detailed blueprints specifying the relative weights of disciplines, organ systems, and competency domains. By integrating these blueprints into the model’s retrieval context, RAG can enforce proportional coverage of item pools, ensuring that generated questions reflect required content distributions [20]. For example, prompts can direct the model to generate exactly 20% internal medicine items, 10% pediatrics, and 5% preventive medicine, mirroring blueprint specifications. In practice, we implement a budgeted generation framework that defines target counts for each blueprint node, followed by systematic reconciliation against the complete blueprint structure. Items exceeding capacity for a particular node are queued for revision or excluded from the pool, while coverage dashboards undergo psychometrician review before pilot testing. This process promotes both quantitative balance and qualitative fidelity to the examination blueprint.

### Curriculum-anchored item generation

RAG also enables curriculum anchoring, allowing the AI to draw directly from validated learning objectives, national clinical guidelines, or authoritative textbooks. This reduces the risk of generating off-scope knowledge or culturally inappropriate content. Grounding items in curricular sources has been shown to enhance both content validity and educator confidence in AI-generated outputs [11]. In the KMLE context, RAG ensures that test items incorporate Korean-specific clinical standards—such as national infectious disease control protocols—that may differ from international norms. To prevent inadvertent rationale leakage, all citations and supporting references are retained in secure internal logs rather than displayed in learner-facing stems or options. Content experts verify that every factual statement within an item stem can be traced to an approved source. When discrepancies arise between international and local guidelines, the most recent national standards take precedence, with alternative perspectives documented in audit logs for transparency.

### Mitigating cultural and geographic bias

Global deployment of LLMs poses a risk of cultural or geographic bias, such as referencing medications not approved in the target jurisdiction. For example, during Peru’s licensing examination, ChatGPT occasionally introduced North American treatment protocols inappropriate for the local context [7]. By constraining AI generation with region-specific formularies and clinical guidelines, RAG minimizes such risks and enhances contextual relevance. We implement strict geographic constraints using localized retrieval indices that include only nationally approved formularies, reimbursement guidelines, and diagnostic standards. Cross-jurisdictional fallback mechanisms are explicitly disabled during retrieval. Any item referencing unsupported medications, dosages, or laboratory reference ranges triggers automatic rejection before reaching expert review. This ensures that all generated items remain both culturally and clinically appropriate for the target testing environment.

### Practical workflow

**Fig. 1.**
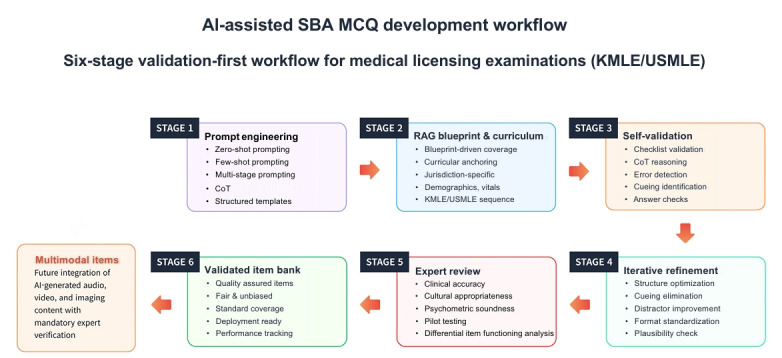
Human-artificial intelligence (AI) workflow for prompt engineering of single best answer (SBA) multiple-choice questions (MCQs) in licensing examinations. The figure outlines a 6-step process: prompt engineering, blueprint alignment with retrieval-augmented generation, AI draft generation, self-validation, expert review, and creation of a validated item bank. Multimodal content can be added with expert oversight. Generative AI supports examiners, but does not replace them. KMLE, Korean Medical Licensing Examination; USMLE, United States Medical Licensing Examination; CoT, chain-of-thought.

In practical application, a RAG-based workflow for SBA generation involves uploading the official blueprint and curriculum, then querying the model with structured prompts (for example, “Generate a pediatrics SBA item on Kawasaki disease aligned with KMLE learning objectives”) (Fig. 1). This process ensures that all generated items are contextually grounded in authoritative references and aligned with curricular scope. We have developed a comprehensive 6-stage operational sequence: (1) systematic ingestion and version control of blueprints, learning objectives, and clinical guidelines into a curated retrieval index; (2) structured retrieve-then-read processing using standardized JSON schema outputs encompassing blueprint nodes, clinical vignettes, lead-in questions, answer options, correct keys, hidden rationales, and source documentation; (3) automated quality checks for scope verification, key-option conflict detection, duplication screening, and cueing heuristic analysis; (4) comprehensive content-expert review with full access to retrieval logs; (5) small-scale pilot testing for psychometric validation; and (6) final item banking contingent upon successful completion of all validation gates. Items that fail scope verification or guideline compliance checks undergo targeted revision with additional retrieval or are excluded entirely to preserve blueprint fidelity.

## SBA MCQ format and style constraints (KMLE case study)

While LLMs can generate clinically relevant multiple-choice items, the validity of licensing examinations depends not only on content accuracy but also on strict adherence to formatting and stylistic conventions. The KMLE provides a particularly detailed case study, as it specifies precise rules for item construction—from vignette structure and option arrangement to typographic presentation [14,15]. Embedding these rules into prompt design is critical for producing usable items. To operationalize these requirements, we systematically translate KMLE formatting standards into structured prompt checklists and automated validation tools, ensuring that all generated items undergo comprehensive formatting assessments before advancing to expert review.

### Lead-in phrasing and avoidance of negatives

SBA items in the KMLE require positively framed stems. Negative phrasing (e.g., “Which of the following is NOT correct?”) is prohibited because it introduces unnecessary cognitive load and increases response error rates [15]. Prompts must therefore explicitly instruct ChatGPT to avoid negative stems and instead formulate concise, direct lead-ins. For example, we systematically transform problematic constructions such as “Which of the following is NOT an appropriate next step?” into clear, positive alternatives such as “What is the best next step?” Automated validation systems identify and reject any stems containing negative qualifiers such as NOT, EXCEPT, or LEAST during the initial screening phase, ensuring compliance with positive phrasing standards.

### Clinical vignette length and structure

KMLE guidelines specify that clinical vignettes follow a standardized sequence: demographics (age, sex), chief complaint, history of present illness, past medical history, physical findings, vital signs, and laboratory results [14]. Prompts must enforce this ordering, as zero-shot generation often omits or reorders key sections. For instance, vital signs should appear in parentheses, formatted as: “blood pressure (BP) 120/80 mm Hg, heart rate (HR) 78/min, respiratory rate (RR) 16/min, T 36.8°C.” We include explicit prompt instructions to maintain concise vignette length (typically 120–150 words), avoid extraneous clinical details, and preserve logical chronological sequencing—from initial presentation through evaluation to diagnosis or management. Any deviation from this structure is automatically flagged for correction before expert review.

### Laboratory values and reference ranges

Another essential constraint involves the standardized notation of laboratory values. The KMLE mandates that reference ranges be included in parentheses and expressed using en dashes or tildes (e.g., “white blood cell [WBC] 7,000/mm^3^ [reference: 4,000–10,000]”) [31]. Embedding rules for laboratory formatting into the prompt helps ensure uniformity. To ensure uniformity, our prompting protocols require strict adherence to the format “analyte value (unit) (reference: low–high),” while explicitly prohibiting the mixing of SI (International System of Units) and conventional units within a single item. An integrated automated unit-checking system identifies inconsistencies or omissions and requires correction before the item proceeds to review.

### Option ordering and distractor design

The KMLE mandates that answer options be ordered from shortest to longest in length to minimize inadvertent clues [15]. Distractors must be homogeneous, clearly incorrect, and free from options such as “all of the above” or “none of the above,” which are explicitly prohibited in the SBA format. These requirements preserve fairness, reduce cognitive bias, and maintain psychometric validity [14]. We further enforce additional design principles: ensuring a single defensible correct answer, maintaining grammatical parallelism across all options, eliminating mutually overlapping or ambiguously correct alternatives, and achieving systematic key-position balance across item sets. Any violation of these principles triggers either automated regeneration or manual editorial intervention. These safeguards collectively uphold the structural and psychometric integrity of generated items.

### Formatting conventions (boxes, bold, and spacing)

The KMLE employs standardized visual formatting conventions, including boxed laboratory data, consistent spacing, and the avoidance of unnecessary bolding or italics. Prompt instructions must therefore capture these typographic standards to prevent inconsistencies that might distract examinees. In our implementation, machine-generated outputs are rendered according to standardized KMLE visual templates, using monospace formatting for laboratory data boxes and uniform paragraph spacing. All textual emphasis (bold, italic, or underlined text) is systematically removed from stems and answer options, while such emphasis is permitted only in internal authoring documentation that remains hidden from examinees.

### Worked example (KMLE-aligned, authoring view; rationales hidden from learners)

Vignette: A 5-year-old boy presents with 5 days of fever and nonexudative conjunctivitis… BP 100/60 mm Hg, HR 110/min, RR 24/min, T 38.6°C. Labs: WBC 12,500/mm³ (reference: 4,000–10,000), CRP 8.2 mg/dL (reference: 0.0–0.5).

Lead-in: Which of the following is the most appropriate next step?

Options (short → long): A. High-dose aspirin; B. Intravenous immunoglobulin; C. Oral amoxicillin for 10 days; D. Oral prednisolone taper; E. Topical erythromycin ophthalmic ointment

Key: B

Rationale (hidden, not shown to learners): Features consistent with Kawasaki disease; IVIG is indicated.

Operational prompt schema (excerpt):

{ "blueprint_node": "Pediatrics—Cardiovascular—Vasculitis—Kawasaki", "vignette": "...", "lead_in": "Which of the following is the most appropriate next step?", "options": {"A":"...", "B":"...", "C":"...", "D":"...", "E":"..."}, "key": "B", "rationale_hidden": "...", "labs_format_ok": true, "option_length_sorted": true, "no_negative_stem": true }

Author checklist (SBA/KMLE): positive lead-in ( ) | KMLE vignette order ( ) | labs with ranges/units ( ) | options A–E homogeneous ( ) | shortest → longest ( ) | single defensible key ( ) | no “all/none of the above” ( ) | rationale hidden ( ) | spacing/boxing per template ( )

## Self-evaluation and quality assurance with ChatGPT

Even when item generation is carefully guided by prompt engineering and blueprint alignment, the final quality of MCQs depends on rigorous evaluation and refinement. Traditionally, human experts have performed quality assurance, but recent advances indicate that ChatGPT itself can be integrated into the validation pipeline, serving as a first-pass reviewer before human oversight. Throughout this review, we maintain a validation-first policy wherein AI-based quality checks act as essential pre-banking gates that complement—but never replace—expert review or pilot statistical analysis. We further require comprehensive audit documentation, including prompts, model versions, reasoning traces, and retrieval sources for every generated item to ensure complete traceability and accountability.

### Checklist-based automated validation

Embedding structured checklists within prompts allows ChatGPT to review its own outputs for compliance. For instance, prompts can instruct the model to verify that: (1) the lead-in is positively phrased, (2) the vignette follows the standardized demographic–history–vital signs–laboratory sequence, (3) reference ranges are included with laboratory values, (4) distractors are plausible yet incorrect, and (5) options are ordered from shortest to longest [27]. This self-validation process mirrors human reviewer checklists and efficiently identifies basic format violations. In practice, we combine rule-based validation scripts with LLM-driven quality checks to systematically detect key-option conflicts, multiple defensible answers, mutually non-exclusive distractors, and unintended cueing signals (e.g., option-length bias or key-position bias). We also perform n-gram and semantic similarity analyses across the entire item bank to prevent content duplication. Items flagged for any violations are automatically rejected or queued for targeted revision prior to expert review.

### CoT for internal reasoning

CoT prompting enables ChatGPT to reveal its reasoning process when evaluating distractor validity and clinical logic consistency. Operationally, CoT frames validation as a sequence of intermediate reasoning steps—such as parsing the vignette, assessing each option, and providing comparative justification—a structure shown to improve model reliability in compositional sub-tasks under few-shot settings [16]. While CoT outputs should never be exposed to examinees, they can be valuable in background quality checks to confirm that the model’s reasoning aligns with sound clinical logic [32]. For example, in a chest pain vignette, CoT reasoning might explain why “acute myocardial infarction” is correct and why “stable angina” or “gastroesophageal reflux disease (GERD)” are plausible but less likely. To preserve assessment security, all CoT traces are stored in secure internal repositories and excluded from learner-facing materials. Expert reviewers use these outputs exclusively to verify logical consistency, confirm source attribution in RAG-based items, and detect any unintended cueing mechanisms.

### Self-refinement and iterative correction

Beyond validation, ChatGPT can perform self-refinement loops. When prompted to identify weaknesses in its own outputs and regenerate improved versions, the model can correct ambiguous lead-ins, strengthen weak distractors, and adjust vignette length [27]. Multi-stage prompting with iterative refinement has been shown to substantially improve compliance with best-practice item writing standards [18]. We employ a systematic 4-stage refinement workflow: (1) automated checking and critique generation, (2) targeted regeneration of only failed components (vignettes, stems, or options), (3) re-evaluation against the full validation checklist, and (4) final expert editing. Each refinement cycle maintains full version control, ensuring traceability, and only items that pass all quality checkpoints proceed to pilot testing.

### Comparison with human review

Despite these advances, AI-based self-evaluation cannot yet replace human judgment [26]. Studies demonstrate that while AI achieves strong structural conformity, human oversight remains indispensable for ensuring clinical accuracy, cultural appropriateness, and psychometric defensibility [10,13]. A hybrid model—employing ChatGPT for first-pass validation followed by expert panel review—emerges as the most efficient and reliable workflow. We therefore define comprehensive minimum standards for human review: each item must be independently evaluated by at least 2 qualified subject-matter experts who approve both content and answer keys, with adjudication procedures for disagreements. Specialized format editors confirm KMLE compliance, while psychometricians conduct pilot analyses of item statistics, including difficulty indices, discrimination parameters, and differential item functioning, before final banking approval.

### Implications for licensing exams

In the context of the KMLE and comparable national licensing assessments, integrating ChatGPT as a validation assistant can reduce faculty workload, improve standardization, and facilitate rapid item bank expansion. However, AI-based validation should always remain complementary, not substitutive, to human psychometric expertise. To ensure operational readiness, we recommend defining explicit go/no-go criteria that require completion of all automated quality checks, consensus approval by subject-matter experts, and satisfactory pilot performance metrics within pre-specified thresholds. Essential security safeguards must include systematic redaction of all CoT reasoning and rationales from learner-accessible materials. Finally, comprehensive governance documentation—including per-item audit trails, version-controlled change logs, and source registries—should be maintained to ensure defensible and transparent decision-making suitable for high-stakes licensing contexts.

## Future directions: hybrid workflows, ethics, and psychometrics

While ChatGPT and related LLMs demonstrate promising capabilities for generating and validating SBA MCQs, their integration into high-stakes licensing examinations requires a forward-looking strategy that balances innovation, reliability, and ethical responsibility. Future developments should prioritize hybrid workflows, robust psychometric evaluation, and explicit ethical safeguards. We therefore outline practical next steps that examination boards can implement immediately while maintaining pathways for future research into more advanced automation approaches.

### Hybrid human-AI workflows

The most sustainable model for examination item development is not full automation but a hybrid pipeline in which AI functions as a drafting and validation tool while human experts provide oversight. Hybrid approaches that combine AI generation with human-approved templates have been shown to enhance both efficiency and item validity [30]. Practical guidelines further emphasize embedding human judgment throughout the process to preserve psychometric rigor [11]. In the KMLE context, this hybrid model could dramatically expand item pools while maintaining alignment with official standards. We recommend implementing a structured division of labor in which AI systems manage initial item drafting and automated checks, while human experts retain authority over final banking decisions. This structure should delineate clear roles among item authors, validators, and psychometricians to minimize confirmation bias, supported by multi-tiered validation gates that include automated quality screening, dual subject-matter expert reviews, and final approval by the examination committee chair. Each item should also maintain a complete audit trail documenting associated prompts, retrieval sources, and version history.

### Psychometric evaluation of AI-generated items

AI-assisted item development and evaluation methods have been examined in a commissioned research report in Korea, which highlighted both feasibility and implementation challenges in national licensing contexts [26]. Even when items adhere to structural guidelines, their psychometric properties must be empirically verified. Pilot testing of AI-generated items is essential to evaluate difficulty indices, discrimination parameters, and distractor functioning [13]. For instance, distractors that appear plausible in theory may fail to attract examinee responses, reducing discrimination. Although AI-assisted simulations of candidate responses may provide preliminary insights, large-scale pretesting remains indispensable for validity and defensibility in licensing contexts. We propose establishing minimum psychometric standards that include pre-registration of analytical protocols, anchor-based equating procedures when combining AI-generated and human-authored items, systematic post hoc differential item functioning analyses across demographic groups, and explicit retirement or revision criteria for items showing statistical drift or security compromise. Only items meeting all predetermined psychometric thresholds should advance to operational item pools.

### Ethical and legal considerations

The integration of AI into assessment raises substantial ethical and legal challenges. Potential risks include plagiarism, propagation of bias, overreliance on AI by educators, and inequities in access to AI-enhanced tools [1]. Furthermore, because most LLMs are trained primarily on English-language data, their outputs may exhibit linguistic bias, leading to less reliable performance in non-English contexts such as the KMLE [20]. From a legal perspective, authorship and intellectual property require clarification. The prevailing consensus is to treat AI strictly as a tool, necessitating transparent disclosure of its role in item generation [10]. We recommend implementing comprehensive documentation protocols that include explicit AI-use statements in all examination materials, systematic provenance logging for all external sources (particularly when employing RAG), privacy-preserving workflows to prevent candidate data exposure in prompts, and stringent security controls to ensure that rationales and CoT reasoning are never visible to examinees. Institutional review boards or ethics committees should provide oversight for any pilot studies involving AI-assisted item development.

### Preparing faculty and institutions

Successful adoption requires systematic training for item writers, examination boards, and faculty to develop AI literacy. Educators must learn not only how to use AI effectively but also how to critically assess its limitations. Recent research in higher education emphasizes “prompt literacy” as an essential competency for responsible integration of LLMs into assessment and evaluation processes [33,34]. Institutional policies should codify best practices for AI use, defining acceptable workflows and accountability mechanisms. We recommend developing a comprehensive training pathway consisting of progressive modules—introductory, authoring, and validation tracks—accompanied by shared prompt libraries tailored to KMLE formatting requirements. These should be supplemented with calibration exercises using standardized item sets and checklist-driven review processes to ensure inter-rater consistency across reviewer cohorts. Building such institutional capacity will be essential for sustainable, ethically grounded integration of AI into examination systems.

## Toward multimedia item generation in national licensing examinations

**Fig. 2.**
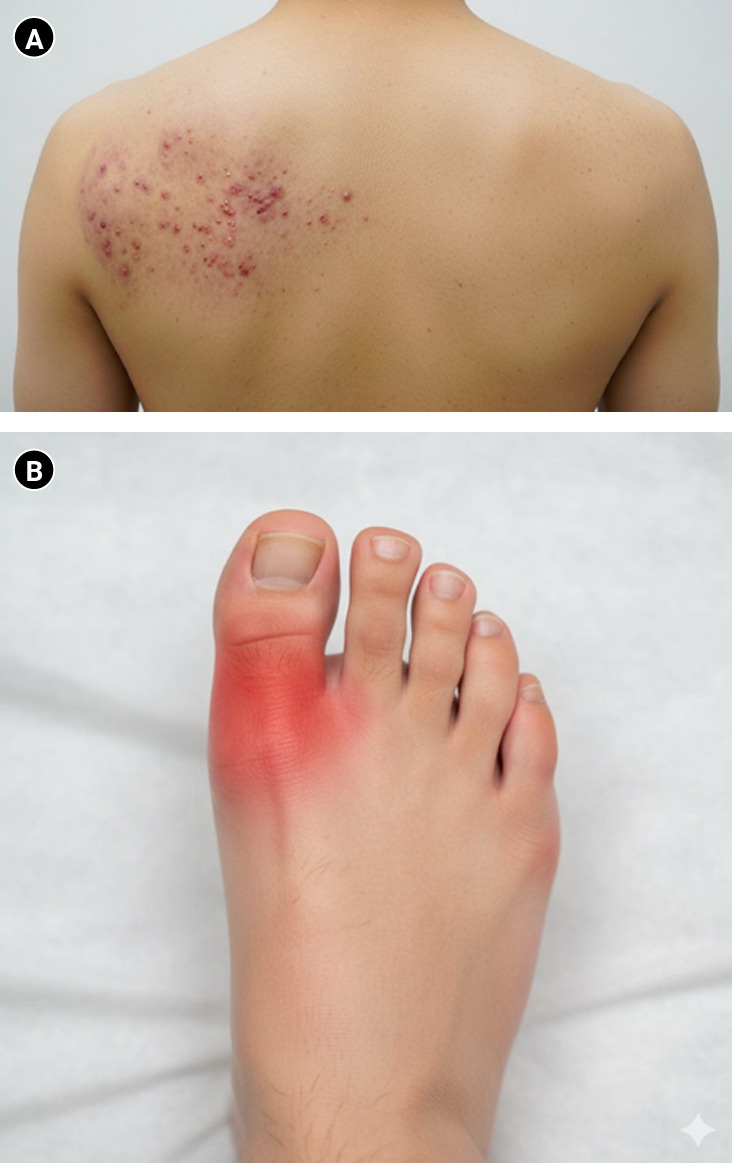
Examples of artificial intelligence (AI)-generated images for medical examination questions. Example clinical images created using generative AI. (A) Herpes zoster skin lesions (Google Whisk) and (B) gouty arthritis of the great toe (Google Imagen).

The format of licensing examinations is evolving beyond static text and still images. In both the United States and Korea, multimedia-based questions incorporating audio (e.g., heart and lung sounds), video clips of physical examination maneuvers, and complex radiological imaging are increasingly integrated into assessments [35]. This trend reflects the growing need to evaluate not only factual recall but also applied clinical reasoning in realistic contexts. Concurrently, rapid advances in controllable image generation and editing technologies—exemplified by developments such as Midjourney, Google Whisk, Imagen, and Gemini 2.5 Flash Image (Nano Banana)—suggest that these tools may soon enable the creation of multimedia materials of sufficient quality for inclusion in national medical licensing examinations (Fig. 2).

### Emergence of multimedia assessments

The incorporation of multimedia items in national examinations reflects the increasing emphasis on authenticity in clinical evaluation. Audio recordings of auscultation findings, video demonstrations of physical signs, and dynamic imaging modalities allow examinations to capture higher-order reasoning and applied decision-making. In both the USMLE and KMLE, the use of multimedia content is gradually expanding, setting a precedent for multimodal testing that more closely approximates real-world clinical encounters [35].

### Advances in GenAI for multimodal content

GenAI has achieved rapid progress in producing high-fidelity images, audio, and video. State-of-the-art text-to-video models such as OpenAI’s Sora and Google’s Veo 2 can generate clinically relevant training clips directly from textual input [36]. In parallel, diffusion-based image generators now produce anatomically accurate and context-specific illustrations, with promising applications in radiology, neurosurgery, and broader medical education [37,38]. Cardiovascular education has particularly benefited from text–image synthesis pipelines that integrate clinical data visualization with physiological explanations [39,40]. These developments are complemented by sophisticated image editing capabilities that enable high-fidelity asset variation and maintain visual consistency from clinician-provided reference images.

### Educational benefits of multimedia generation

AI-enabled multimedia has the potential to standardize testing experiences by ensuring consistent quality across audio clips, imaging displays, and procedural videos. Such innovations could reduce examiner variability, enhance fairness, and improve construct validity. Moreover, dynamic scenarios—such as videos depicting seizure episodes or audio recordings of abnormal breath sounds—offer greater ecological validity by simulating real clinical situations [41].

### Risks of inaccuracy and the need for expert review

Despite these opportunities, AI-generated multimedia introduces new risks. Studies have shown that even highly realistic outputs can contain subtle anatomical or pathological inaccuracies that may mislead learners if left unverified [37,42]. Similarly, generated audio and video clips may exhibit artifacts that distort diagnostic cues or reduce clinical authenticity. To mitigate these risks, systematic expert validation by domain specialists is essential. Human oversight ensures that multimedia items meet licensing standards and preserve both fairness and content validity [12].

### Implications for national licensing examinations

The growing adoption of multimedia formats in examinations such as the USMLE and KMLE suggests that integration of GenAI into this domain is both inevitable and transformative. Properly implemented, AI can accelerate item development, expand multimedia banks, and reduce faculty workload. However, such adoption must occur within a human-in-the-loop framework that ensures medical accuracy, psychometric rigor, and ethical responsibility. Global sharing of templates, metadata standards, and validation protocols could further promote harmonization of best practices, supporting defensible and transparent use of multimedia AI across licensing systems [39]. For near-term implementation, we recommend restricting AI applications to asset drafting, controlled editing, and variant generation, while maintaining rigorous expert oversight for clinical accuracy verification, compliance with jurisdiction-specific standards, and comprehensive pilot validation before operational deployment.

## Conclusion

The integration of LLMs into the development of licensing examination items represents both an unprecedented opportunity and a profound responsibility. Evidence from multiple national examinations demonstrates that LLMs such as ChatGPT can already perform at or near passing thresholds and show potential as grading assistants. Yet answering existing questions and generating new ones are qualitatively distinct challenges. The creation of valid SBA items demands strict adherence to psychometric principles, blueprint alignment, and formatting rules established by licensing authorities. Accordingly, we position GenAI as a human-in-the-loop assistant that augments rather than replaces expert-driven item development.

This review has outlined strategies to enhance AI-generated item quality. Prompt engineering, encompassing zero-shot, few-shot, multi-stage, and CoT methods, improves structural fidelity and clinical plausibility, while RAG ensures blueprint and curricular alignment. Concurrently, checklist-based self-validation, iterative refinement, and AI-assisted quality assurance illustrate how LLMs can function as first-pass reviewers under expert supervision. Central to this framework is the principle that rationales and CoT outputs remain strictly internal to the authoring process and are never included in learner-facing materials. Comprehensive pre-banking validation—including automated checks, subject-matter expert review, and pilot testing—remains essential for defensibility.

The broader future of AI in assessment lies in hybrid human–AI workflows. While generative models can efficiently draft and validate questions, human experts remain indispensable for ensuring clinical accuracy, cultural appropriateness, and psychometric soundness. Ethical and legal issues—including bias, plagiarism, authorship, and equitable access—must also be proactively managed. Preparing faculty through AI and prompt literacy training, supported by institutional policies and transparent governance, is essential for responsible adoption. We further recommend explicit AI-use disclosures in all examination documentation, comprehensive provenance logging for all generated or retrieved materials, and robust privacy protections to prevent rationale exposure and safeguard examinee confidentiality.

Looking forward, the expansion of multimedia assessment introduces both opportunities and challenges. Advances in generative imaging, audio, and video technologies could support the creation of authentic, multimodal clinical scenarios. However, expert verification is non-negotiable: even subtle inaccuracies in medical visuals, sounds, or videos may undermine test validity. Until substantial empirical evidence supports full operational use, AI-generated multimedia should remain limited to expert-verified, well-documented assets that demonstrate satisfactory pilot performance before inclusion in active item pools.

Taken together, these developments define a roadmap for the responsible integration of GenAI in high-stakes assessment. Hybrid pipelines combining AI efficiency with human judgment—grounded in psychometric evidence, ethical safeguards, and regulatory compliance—can expand item pools and enhance standardization. Rather than replacing experts, ChatGPT and related models should be recognized as transformative assistants that, when properly governed, can advance the rigor, fairness, and efficiency of health professions assessment. This practice-oriented roadmap, spanning strategic prompt design, RAG-based blueprint alignment, validation-first governance, and KMLE-specific formatting, offers an implementable and methodologically defensible framework for the next generation of licensing examinations.

## Figures and Tables

**Figure f3-jeehp-22-34:**
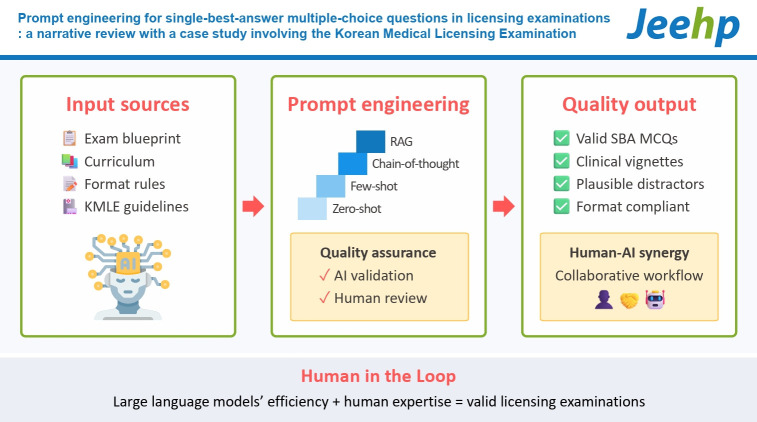


**Table 1.** Summary of item-development approaches

Approach	Characteristics	Advantages	Disadvantages	Concrete examples
Zero-shot prompting	Model generates multiple-choice questions without examples.	Simple, fast; diverse coverage (recall, reasoning, problem-solving).	Often deviates from required formats; implausible distractors; inconsistent option ordering.	Used only for initial ideation, followed immediately by structural checklists and automated cueing detection.
Few-shot prompting with exemplars	Supplies 3–5 validated single-best answer items before generation.	Improves structural conformity, distractor plausibility, and clinical realism; reproduces tone/length; adapts to exam conventions.	Requires high-quality, curated exemplars.	KMLE-aligned sequence (demographics → chief complaint → history → exam → labs); improves cultural and linguistic appropriateness.
Multi-stage prompting	Stepwise process: topic → vignette → lead-in → options → rationale → self-critique.	Reduces logical errors and answer leakage; enables iterative correction/self-refinement.	Adds workflow complexity.	Six-stage schema with blueprint alignment, vignette drafting, option balancing, rationale logging, and automated self-critique before expert review.
CoT prompting	Explicit reasoning steps during generation or validation.	Improves few-shot item generation performance; strengthens logical consistency and distractor plausibility.	Rationales must be redacted from learner-facing materials.	Example: Kawasaki vignette with IVIG as correct key; reasoning retained internally for validation only.
Structured formats (markdown/JSON)	JSON schemas for field consistency; markdown rubrics for readability.	Substantially improves accuracy and structure; enables systematic validation.	Requires upfront schema design.	JSON schema with fields (blueprint node, vignette, options A–E, key, rationale_hidden); markdown used for evaluation rubrics.
RAG	Grounds prompts in blueprints, curricula, guidelines.	Enforces proportional coverage; reduces scope drift; ensures cultural and jurisdictional alignment.	Requires curated retrieval index.	Aligns items to KMLE blueprint weights; enforces Korean guidelines and reference ranges.
Self-evaluation/self-refinement	Checklist-based validation and iterative correction.	Improves clarity, vignette structure, distractor quality, and option ordering.	Cannot replace human review; still requires expert oversight.	Model critiques and regenerates vignettes or options, re-evaluates, then passes to expert editing.
Hybrid human-AI workflow	AI drafts and validates; humans finalize and bank items.	Scalable; reduces faculty burden; preserves psychometric rigor.	Still requires dual expert review, pilot testing, and governance protocols.	Division of labor: AI drafts single-best answer pool, human experts verify clinical accuracy, style, and psychometrics before banking.

KMLE, Korean Medical Licensing Examination; CoT, chain-of-thought; IVIG, intravenous immunoglobulin; JSON, JavaScript Object Notation; RAG, retrieval-augmented generation; AI, artificial intelligence.

**Table 2.** Prompting selection guide for multiple-choice question authors

Author goal	Recommended strategy	Key advantages	Precautions/validation requirements
Brainstorming new item ideas	Zero-shot prompting	Generates diverse clinical scenarios quickly.	Use only for ideation; filter with structural checklists and automated cueing detection.
Producing format-fidelity single-best answer items	Few-shot prompting with high-quality exemplars	Improves structural conformity, distractor plausibility, tone, and length.	Exemplars must strictly follow local exam rules (e.g., KMLE vignette order, lab notation).
Reducing logical errors and answer leakage	Multi-stage prompting/CoT prompting	Strengthens reasoning consistency; prevents leakage; enables iterative correction.	Rationales/CoT reasoning must remain hidden from learner materials.
Ensuring blueprint and curriculum alignment	Retrieval-augmented generation	Guarantees proportional coverage and jurisdiction-specific scope.	Requires curated retrieval index; all content must be verified against authoritative sources.
Automating pre-review checks	Structured JSON/Markdown + AI self-validation	Enforces uniform schema; supports efficient checklist-based validation.	Complements, but cannot replace, human review.
Enhancing distractor quality and option balance	Iterative self-refinement loops	Improves plausibility, grammatical consistency, and option ordering.	Requires systematic re-validation and expert editing.
Scaling defensibly	Hybrid human-AI workflow	Expands item pools efficiently with defensible audit trails.	Requires dual expert review, psychometric testing, and adherence to governance frameworks.

KMLE, Korean Medical Licensing Examination; CoT, chain-of-thought; JSON, JavaScript Object Notation; AI, artificial intelligence.

## Supplementary materials

Supplement 1. Example prompt for custom GPT instruction for single-best-answer questions for the Korean Medical Licensing Examination (KMLE).

Supplement 2. Audio recording of the abstract.
